# Histological improvement in chronic hepatitis B patients treated with bicyclol: real world experience

**DOI:** 10.1186/s12876-019-1005-1

**Published:** 2019-06-13

**Authors:** Xiaoling Chi, Huanming Xiao, Meijie Shi, Gaoshu Cai, Yubao Xie, Junmin Jiang, Guangjun Tian, Shuduo Wu, Chaozhen Zhang, Pengtao Zhao, Jiezhen Chen

**Affiliations:** 1 Department of Hepatology, Guangdong Provincial Hospital of Chinese Medicine, The Second Affiliated Hospital of Guangzhou University of Chinese Medicine, 111 Dade Road, Guangzhou, 510120 Guangdong Province China; 2 Guangzhou University of Chinese Medicine, Guangzhou, 510403 Guangdong Province China

**Keywords:** Bicyclol, Chronic hepatitis B, Hepatoprotective drug, Liver biopsy

## Abstract

**Background:**

Bicyclol, the most commonly-used liver hepatoprotective drug in China, is often selected to control disease progression in CHB patients who refuse anti-viral treatment. However, data on histological changes after bicyclol treatment in these patients are scarce. Therefore, this study has been conducted to find out whether bicyclol has good benefits of histological improvement in CHB patients who refuse anti-viral agents.

**Methods:**

The demographic, clinical and pathological data were collected from CHB patients who received bicyclol from January 2010 to June 2016. Improvement in liver inflammation or fibrosis is defined as at least one-grade or one-stage decrease as measured by the Scheuer scoring system. Thirty patients treated with ETV for 48 weeks were chosen as a control group to compare the histological improvement between bicyclol and entecavir (ETV) after 48-week treatment.

**Results:**

A total of 123 patients with CHB treated with bicyclol were included in this study. Paired liver biopsies were performed in 70 patients. Inter-biopsy interval was 17.44 ± 8.90 months (12–60 months). As shown by facts, 41.4% patients achieved liver inflammation improvement, while only 10.0% patients showed liver inflammation progression after bicyclol treatment. In regarding to liver fibrosis, as shown by facts, 28.6% patients achieved fibrosis improvement. More importantly, It was found that the proportions of patients with liver inflammation and fibrosis improvement were both not significantly lower than those in ETV group (53.3% vs 63.3 and 36.7% vs 43.4%). Most of patients (82.4%) with elevated baseline ALT became normal after bicyclol treatment. More importantly, as shown by the multi-variate analysis, the treatment course of bicyclol was an independent factor for liver inflammation improvement. With the HBeAg status adjusted, ALT and HBV-DNA quantity, the odds ratio (95% confidence interval) of patients with ≥48-week treatment was 5.756 (1.893,17.500) when compared with patients via < 48-week treatment.

**Conclusion:**

Bicyclol can improve liver inflammation and the ALT normalization rate of CHB patients, especially when the treatment course is prolonged. This has confirmed that bicyclol could control hepatitis activity, which might be a good choice for CHB patients who refuse anti-viral treatments.

## Background

Chronic hepatitis B (CHB) is caused by hepatitis B virus (HBV) infection, while 15–40% of patients with CHB can develop liver cirrhosis and hepatocellular carcinoma (HCC) [1]. Results from the Reveal-HBV study group [2] suggest that the elevated serum HBV-DNA level is an independent risk predictor of liver cirrhosis or HCC. Thus, anti-viral treatment with interferon (IFN) or nucleos(t)ide (NUC) analog has been strongly recommended by previous treatment guidelines [3–5] to prevent progression of CHB to cirrhosis, HCC, and even death. However, some shortcomings of these anti-viral agents are still in existence. For example, patients treated with IFN/pegylated IFN might suffer from intolerable adverse effects, while the long-term therapy of NUC may result in high costs or strong drug resistance. Therefore, not all patients with CHB, who need antiviral treatment according to the guidelines, can accept antiviral treatment. In China, some of these patients might seek for hepatoprotective drugs or Chinese medicine instead.

Liver cells are constantly attacked by HBV, and continuous liver inflammation, the basic pathologic feature of CHB progression, might induce liver fibrosis or cirrhosis [6]. Therefore, effective control over hepatitis activity might improve the long-term outcome of patients with CHB. In China, a large number of studies on hepatoprotective and antiviral drugs have been conducted to find some useful drugs to control the hepatitis activity in patients with CHB. Researchers have realized the importance of hepatoprotective drugs in the treatment of patients with CHB, especially in patients who refuse anti-viral treatment or still have abnormal alanine aminotransferase (ALT) level during anti-viral treatment [7]. Bicyclol is a hepatoprotective drugpolpularly used in China, showing positive efficacy in patients with CHB. Both previous experimental and clinical studies [8, 9] have shown some good effects, such as anti-liver injury, anti-liver inflammation, and anti-liver fibrosis, and so on. HBeAg positive CHB was treated with adefovir dipivoxil (ADV) plus bicyclol for 48 weeks. The serum aminotransferase level and the Knodell score in ADV plus bicyclol combination therapy group decreased greater than those in ADV monotherapy group.(*P* < 0.01, *P* < 0.05) [10]. Thirty-one patients with chronic viral hepatitis B were treated with bicyclol for 36 weeks, there were significant differences in the index of histological activities. Bicyclol tablets are effective in improving liver histological changes in chronic hepatitis B patients [11]. A retrospective cohort study, including patients from the hospital information system (HIS; established by the Chinese Academy of Medical Sciences) viral hepatitis database is composed of 18 third-grade class A hospitals in China, the findings indicate that Bicyclol tablets can improve the ALT normalization rate of CHB patients showing mild ALT elevation [12]. However, most previous clinical studies focused on evaluating the ALT normalization rate, hepatitis B e antigen (HBeAg) seroconversion rate, or changes in HBV-DNA levels after bicyclol treatment. Clinical data on significant histological improvement in patients with CHB treated with bicyclol are scarce. Therefore, this study has been conducted to find out whether bicyclol has good effects of histological improvement on patients with CHB who refuse anti-viral agents.

## Methods

### Study population

Eligible patients were selected from patients with CHB hospitalized in Guangdong Provincial Hospital of Chinese Medicine between January 2010 and June 2016 according to the following inclusion and exclusion criteria.

The inclusion criteria are as follows: (1) those aged from 18 to 65 years, (2) with a diagnosis of CHB according to the criteria described in the Guideline of Prevention and Treatment for CHB (2010 version) [4], (3) with significant liver inflammation (*G* ≥ 2) shown by the first liver biopsy or ALT ≥2 × upper limit of normal, (4) having refused antiviral treatment but received bicyclol treatment instead according to personal wishes of patients, (5) written informed consents obtained from all patients before enrollment. An interbiopsy interval duration of liver biopsy should be at least 1 year in the patient who has received paired liver biopsy. The progression of liver disease was assessed based on the findings of the second liver biopsy. All eligible patients were followed up in the outpatient department integrated with email and telephone until the second liver biopsy for patients maintained on bicyclol treatment, or the beginning of anti-viral therapy for the patients who discontinued bicyclol treatment due to the withdrawal or failure of treatment.

The exclusion criteria are as follows: (1) co-infection with hepatitis A virus, hepatitis C virus, hepatitis D virus, hepatitis E virus, or human immunodeficiency virus; (2) concomitant liver and gallbladder diseases including evident advanced primary biliary cirrhosis, autoimmune hepatitis, decompensated cirrhosis, severe hepatitis, or hepatic carcinoma and a history of excessive alcohol consumption (20 g/day for females and 30 g/day for males); (3) serious uncontrollable heart, kidney, lung, endocrine, blood, metabolic, or gastrointestinal primary diseases, or mental illness; and (4) having received any other hepatoprotective drug or anti-viral treatment within 6 months prior to the enrollment.

In order to compare the histological improvement between bicyclol and entecavir (ETV) after 48-week treatment, 30 patients treated with ETV in a study registered in 2012 were chosen as a control group to match the the treatment group in a 1:1 ratio by age, gender, HBeAg status and HBV-DNA level.

This study has been approved by the Ethics Committee of the Guangdong Provincial Hospital of Chinese Medicine and carried out in accordance with the Declaration of Helsinki. Written informed consents were obtained from all patients during the outpatient follow-up or by telephone and letter follow-up before the enrollment.

### Clinical and laboratory assessment

The demographic, clinical, and laboratory data at the time of liver biopsy were collected from medical records, including the age, gender, levels of ALT, aspartate aminotransferase (AST), and viral parameters. The levels of serum hepatitis B surface antigen (HBsAg) and HBeAg were measured by using electrochemical immunoassay (Elecsys 2010, Roche Diagnostics, Mannheim, Germany), and the serum HBV-DNA level with a lower limit of detection of 500 IU/mL was measured by using ABI 7300 (Applied BiosystemsInc, NYC, New York, USA). The findings of abdominal B ultrasound or upper abdominal computed tomography or magnetic resonance imaging were also recorded.

### Histological assessment

A liver biopsy was performed by using 18G MAXCO needles (Bard Co., NJ, USA). Specimens were fixed in 10% formalin, embedded in paraffin, and stained with hematoxylin and eosin. A minimum of 15-mm length of liver tissue and at least 10–15 portal tracts were required for the diagnosis. Pathologic specimens were analized by two experienced histopathologists of our hospital in double-blind manner. The liver necroinflammatory activity and fibrosis were each scored on a 0–4 scale by using the Scheuer scoring system [13]. Improvement or progression of necroinflammation was defined as at least one-grade lower or higher than the base-line in the necroinflammatory activity. Improvement or progression of fibrosis was defined as at least one-stage lower or higher than the baseline in the fibrosis too, whereas no change or less than one-grade or one-stage change relative to the baseline score was identified as the absence of progression.

### The therapeutic schedule

All the enrolled patients received 75 mg (25 mg, tid) of bicyclol (Beijing Union Pharmaceutical Factory, Beijing, China) daily for at least 12 weeks. Patients with other anti-viral drugs or hepatoprotective drugs during the therapy should be excluded.

### Statistical analysis

Continuous variables were expressed as the mean and standard deviation, or median and interquartile range, and compared by using the Student *t* test or nonparametric test (Wilcoxon) as being appropriate. Categorical variables were presented as counts and percentages. Categorical parameters among groups were compared by using the chi-square test. The causative factors of improvement in liver inflammation were determined by making analysis of univariate and multivariate logistic regression. All *P* values were two tailed. A *P* value of less than 0.05 was considered statistically significant. All statistical analyses were made by using the SPSS software version 19.0 (SPSS Inc., IL, USA).

## Results

### Base-line characteristics of patients

A total of 232 CHB patients treated with bicyclol were initially screened in this study. However, 109 patients were excluded for some reasons, such as the combination with other antiviral drugs or hepatoprotective drugs, or incomplete data, etc. Finally, 123 patients with CHB treated with bicyclol tablets were included in this study. The median duration of bicyclol therapy lasted 48 weeks (12-232 weeks). The median age of the patients was 35 years and most of them were males (69.1%). Paired liver biopsies were performed in 70 patients. Inter-biopsy interval was 17.44 ± 8.90 months (12–60 months). Of these 70 patients, 40 patients were treated with < 48-week bicyclol treatment, while 30 patients treated with ≥48-week bicyclol treatment. Table 1 lists detailed information of the enrolled patients with CHB.

**Table 1:** Baseline characteristics of 123 CHB patients treated with bicyclol tablets

Variables	All patients (*n* = 123)	Normal ALT (*n* = 38)	Elevated ALT (*n* = 85)
Demographic characteristics			
Median age, years (range)	35 (18–65)	39 (23–61)	33 (18–65)
Males, n (%)	85 (69.1)	25 (65.8)	60 (70.6)
Laboratory data			
Median ALT, U/L (range)	68.0 (5–483)	24.5 (5–50)	98 (51–483)
Median AST, U/L (range)	44.0 (15–323)	23.0 (15–40)	56 (27–323)
HBeAg (+), n (%)	66 (53.7)	9 (23.7)	57 (67.1)
HBV DNA (mean ± SD), log10IU/ml	5.99 ± 1.76	4.43 ± 1.79	6.67 ± 1.24
Liver histology			
Inflammation activity, n (%)			
G2, n (%)	61 (87.1)	20 (80.0)	41 (91.1)
G3, n (%)	9 (12.9)	5 (20.0)	4 (8.9)
Fibrosis (%)			
S1, n (%)	22 (31.4)	7 (28.0)	15 (33.3)
S2, n (%)	37 (52.8)	11 (44.0)	26 (57.8)
S3, n (%)	11 (15.8)	7 (28.0)	4 (8.9)

### Biochemical improvement and virological and serological responses in patients after bicyclol treatment

As shown in Table 2, many patients (82.4%) with elevated baseline ALT level became normal after bicyclol treatment. 16.7% of patients showed HBeAg loss after bicyclol treatment. And the HBV-DNA levels were only found to be significantly decreased from the base-line (from 6.67 to 5.76log10 IU/mL, *P* < 0.001) in patients with elevated base-line ALT level.

**Table 2:** Biochemical, virological, serological responses and APRI change after bicyclol treatment

variable	All patients (*n* = 123)	Normal ALT (*n* = 38)	Elevated ALT (*n* = 85)
ALT≤1 × upper limit of normal, n(%)	107 (87.0)	37 (97.4)	70 (82.4)
HBeAg loss, n(%)	11 (16.7)	2 (22.2)	9 (15.8)
HBV DNA (mean ± SD), log10IU/ml			
Pre-treatment	5.99 ± 1.76	4.43 ± 1.79	6.67 ± 1.24
Post-treatment	5.20 ± 1.77^*^	4.23 ± 1.66	5.76 ± 1.59^*^

*Compared to pretreatment level, *P* < 0.05

### Histological improvement

**Fig. 1:**
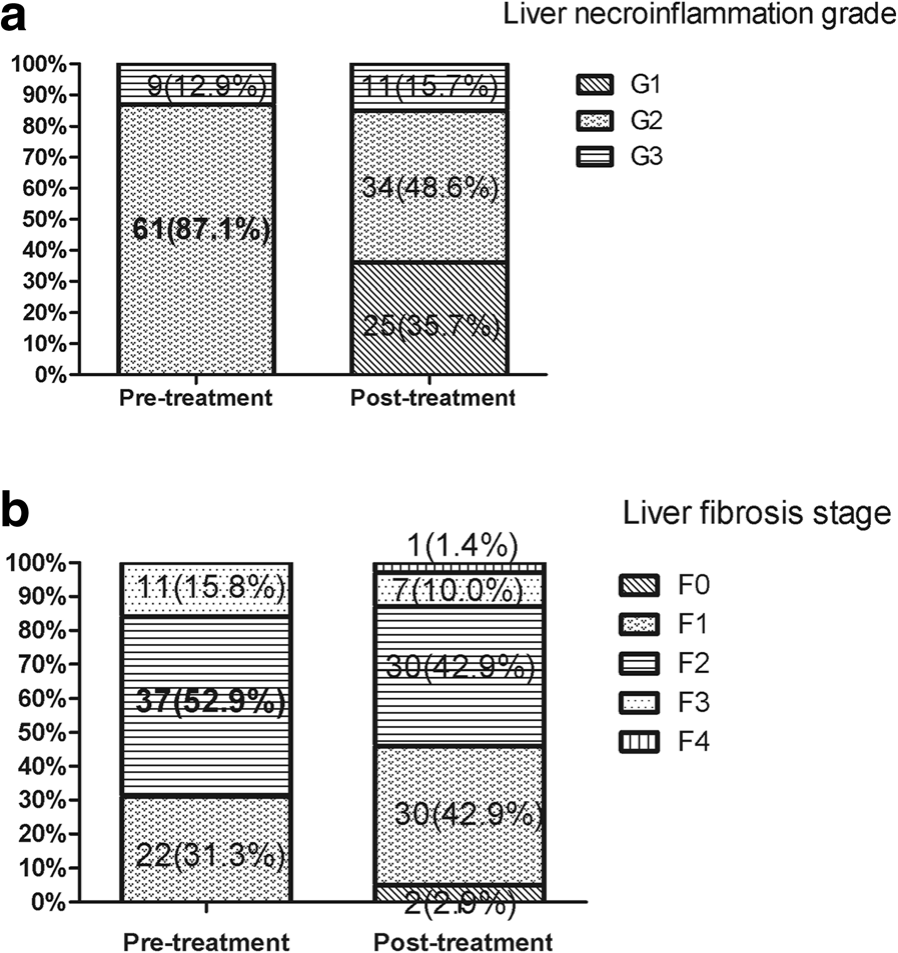
Changes of liver inflammation and fibrosis after bicyclol treatment. Both liver inflammation grade and fibrosis stage were decreased after bicyclols treatment. The improvement seemed more significant in the liver inflammation grade. The number of patients with significant liver inflammation (G2 or G3) decreased to 45 (64.3%) from 70 (100%) at the base-line. **a.** 41.4% (29/70) patients achieved liver inflammation improvement. **b. **28.6% (20/70) patients achieved fibrosis improvement

**Fig. 2:**
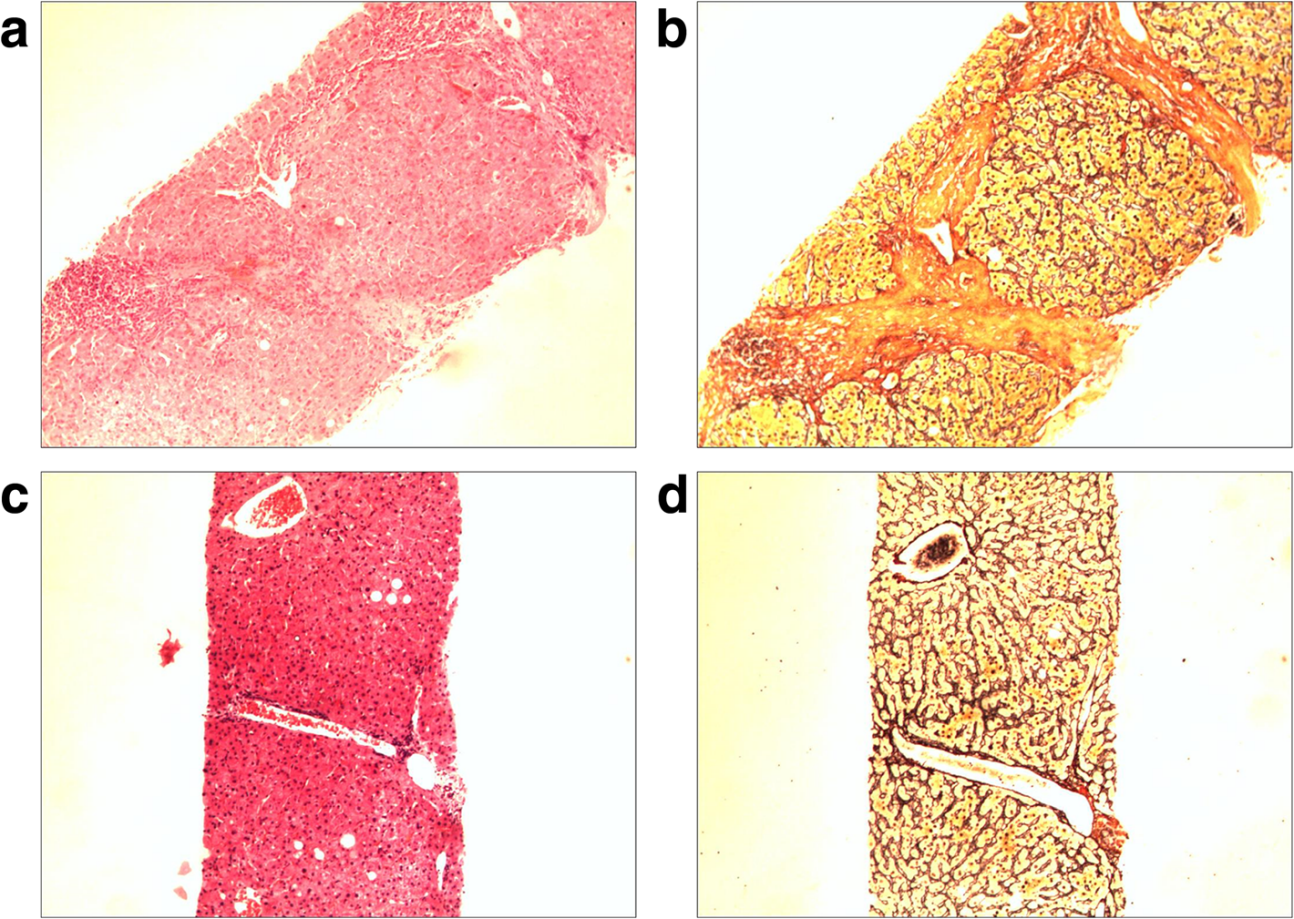
Changes in liver histology before and after treatment (× 100). (Male, age = 38 years, the course of treatment = 48 weeks). **a**. HE staining before treatment: Moderate to massive punctate and patchy necrosis inside the hepatic lobule, moderate lymphocyte infiltration into the portal tracts, and moderate interface inflammation with portal–portal bridging necrosis. **b**. Warthin–Starry staining before treatment: Fibrosis of the necrotic zones of the portal tracts, fibrotic collapse of the necrotic zones, and moderate formations of spiky fibers and fibrotic septa, leading to hepatic lobular structural deformation; typical nodule not found. Diagnosis: Chronic hepatitis, moderate (G3S3). **c**. HE staining after treatment: Mild punctate and patchy necrosis inside the hepatic lobule, small amount of lymphocytes in the portal tracts, and no interface inflammation. **d**. Warthin–Starry staining after treatment: Fibrosis of the portal tracts with sporadic spiky fibers. Diagnosis: Mild lesion (G1S1)

After bicyclol treatment, 41.4% patients achieved liver inflammation improvement, while only 10.0% patients showed liver inflammation progression after bicyclol treatment (Fig. 1a). With regard to liver fibrosis, as shown by facts, 28.6% patients achieved fibrosis improvement and 14.3% patients showed fibrosis progression (Fig. 1b). None of the patients progressed into cirrhosis. More importantly, liver inflammation improvement in patients with bicyclol of more than 48 weeks was significantly higher than that in patients with bicyclol of less than 48 weeks (69.23% vs 25.00%, *P* = 0.001). Figure 2 showed photomicrographs of biopsy samples taken from one patient, a 38-year-old male with base-line inflammation grade and fibrosis stage of 3 and 3, respectively. After 48-week bicyclol treatment, both his liver inflammation grade and fibrosis stage decreased to 1 and 1.

Moreover, there was no significantly difference in histological changes between bicyclol group and ETV group after 48-week treatment. The proportion of patients showed liver inflammation improvement in bicyclol group (53.3%, 16/30) was not significantly lower than that in ETV group (63.3%,19/30, *P* = 0.432). Meanwhile, 36.7% (11/30) patients showed liver fibrosis improvement, which was no significantly lower than that (43.4%, 13/30) in the control group (*P* = 0.598). Only 6.7% (2/30) patients showed inflammation progression in bicyclol group, which was also no significantly higher than that in ETV group (3.3%, 1/30, *P* = 0.680). More detailed information between these two groups can be seen in Table 3.

**Table 3:** Histological changes between bicyclol group and ETV group after 48-week treatment

variables	Bicyclol group (*n* = 30)	ETV group (*n* = 30)
Inflammation activity, n (%)		
Inflammation improvement	16 (53.3)	19 (63.3)
No change	12 (40.0)	10 (33.4)
Inflammation progression	2 (6.7)	1 (3.3)
Liver Fibrosis, n (%)		
Fibrosis improvement	11 (36.7)	13 (43.4)
No change	16 (53.3)	16 (53.3)
Fibrosis progression	3 (10.0)	1 (3.3)

### Related factors of improvement in liver inflammation after bicyclol treatment

As indicated by results of univariate analysis,the treatment course of bicyclol and base-line ALT level significantly affected the liver inflammation improvement, while other factors, such as age, sex, HBeAg status, HBV-DNA quantity had little impact on the outcome (Table 4). More importantly, as shown by multivariate analysis, the long treatment course of bicyclol (≥48 weeks) was an independent factor for liver inflammation improvement. With the HBeAg status adjusted, ALT and HBV-DNA quantity, the odds ratio (95% confidence interval) of patients with ≥48-week treatment was 5.756 (1.893,17.500) when compared with patients with < 48-week treatment (Table 5).

**Table 4:** Monofactorial analysis of liver inflammation improvement

Factor	I Improved *n* (%)	Not improved *n* (%)	*P*	Unadjusted OR (95%CI)
Treatment course of bicyclol (weeks)			0.001	6.750 (2.300, 19.811)
≥ 48	21 (70.00)	9 (30.00)		
< 48	10 (25.00)	30 (75.00)		
Age (years)			0.211	2.639 (0.577, 12.064)
≥ 50	5 (62.50)	3 (37.50)		
< 50	24 (38.71)	38 (61.29)		
Sex			0.176	2.816 (0.705, 6.777)
Female	9 (56.25)	7 (43.75)		
Male	20 (37.04)	34 (62.96)		
ALT (U/L)			0.024	0.301 (0.107, 0.851)
≥ 40	15 (31.91)	32 (68.09)		
< 40	14 (60.87)	9 (39.13)		
HBeAg			0.184	1.923 (0.733, 5.043)
Negative	16 (50.00)	16 (50.00)		
Positive	13 (34.21)	25 (65.79)		
HBV-DNA			0.341	0.532 (0.146, 1.948)
≥ 10^5^ IU/mL	23 (38.98)	36 (61.02)		
< 10^5^ IU/mL	6 (54.55)	5 (45.45)

**Table 5:** Multifactorial analysis of liver inflammation improvement

Factor	Wald Chi-square	P	Adjusted OR (95% CI)
Treatment course of bicyclol (≥48 weeks or < 48 weeks)	9.517	0.002	5.756 (1.893,17.500)
ALT (≥40 U/L or < 40 U/L)	1.779	0.182	0.395 (0.101,1.546)
HBeAg (negative or positive)	0.480	0.489	1.519 (0.465,4.957)
HBV-DNA (≥10^5^ IU/mL or < 10^5^ IU/mL)	0.311	0.577	1.652 (0.283,9.648)

### Safety of treatment

Based on the safety data collected throughout the study, no severe adverse events occurred during the process. Only seven patients with elevated ALT showed abnormal ALT after irregular withdrawal of bicyclol during the therapy.

## Discussion

Bicyclol, the most commonly used liver hepatoprotective drug in China, is often selected to control disease progression in CHB patients who refuse anti-viral treatment. However, data on histological changes after bicyclol monotherapy treatment in these patients are scarce. Therefore, we conducted this study to find out whether bicyclol has good effects on CHB patients, such as histological improvement or biochemical improvement or virological response and so on.

With regard to histological changes after bicyclol treatment, it showed that no patient had progressed to cirrhosis or liver cancer in the second liver biopsy. Moreover, 41.4% (29/70) of patients achieved inflammation improvement, while 10.0% (7/70) of patients showed inflammation progression. At the same time, ALT changes are similar to the results of previous studies [14, 15]. Most of patients (82.4%) with elevated baseline ALT became normal after bicyclol treatment. However, only 28.6% (20/70) of patients achieved fibrosis improvement. These results have confirmed the anti-liver inflammation effect of bicyclol, while having shown a relative limited anti-fibrosis effect. It might be due to the limited patients who accept the long term treatment of bicyclol and many patients who are not taking bicyclol for a longer period during the liver-biopsy interval. Therefore, in order to explore the efficacy of bicyclol more accurately, histological improvement was compared between bicyclol group and ETV group after 48-week treatment. It was found that the proportions of patients with liver inflammation and fibrosis improvement were both not significantly lower than those in ETV group (53.3% vs 63.3 and 36.7% vs 43.4%). These histological changes were similar to those of previous research [16], which revealed that after one-year NUC therapy for CHB, hepatic inflammation decreased in 50 to 70% of patients, while fibrosis frequently remained unchanged. This has confirmed that bicyclol has good effects on liver inflammation improvement, which might be a good choice for CHB patients who refuse antiviral treatments. In terms of serology and virology, HBeAg loss took place in 16.7% of patients after bicyclol treatment. The decrease of HBV-DNA was statistically significant. Which indicated that bicyclol may play a considerable role in anti-virus while resisting inflammation and protecting the liver [10, 17].

As for related factors of liver inflammation improvement, the multivariate analysis showed that the treatment course of bicyclol was an independent factor for liver inflammation improvement. The longer of the treatment course, the more significant the improvement in liver inflammation. With the HBeAg status adjusted, ALT and HBV-DNA quantity, the odds ratio (95% confidence interval) of patients with ≥48-week treatment was 5.756 (1.893,17.500) when compared with patients with < 48-week treatment. Therefore, some famous experts [18] recommended clinical doctors to prolong the treatment course of bicyclol, as a promising approach to achieve a desirable effect of anti-liver inflammation.

In safety analysis, no severe adverse event occurred during the treatment process, suggesting the good safety profile of bicyclol. However, it is worth mentioning that seven patients with elevated ALT showed abnormal ALT after irregular withdrawal of bicyclol during the therapy. Therefore, bicyclol should be withdrawn gradually. Also, it is quite important to increase the patient compliance.

However, a potential limitation of this study is that it was a single-center study and the number of enrolled patients was rather limited, although it was merely for the proof of real-world experience. Therefore, further large and prospective studies with a long-term follow-up should be conducted to confirm the histological improvement after bicyclol treatment.

## Conclusions

In summary, bicyclol can improve liver inflammation and the ALT normalization rate of CHB patients, especially when the treatment course is prolonged. This has confirmed that bicyclol can control hepatitis activity, which might be a good choice for CHB patients who refuse anti-viral treatment. We also suggest that don’t interrupt the treatment casually. Because of the limited number of patients, more clinical trials are still needed to verify the histological improvement of bicyclol in patients with CHB who refuse anti-viral treatment.

## Data Availability

The datasets supporting the conclusions of the current study are available at Guangdong Provincial Hospital of Chinese Medicine, which are available from the corresponding author on reasonable request.

## Abbreviations

ALT: Alanine aminotransferase
AST: Aspartate aminotransferase
CHB: Chronic hepatitis B
ETV: Entecavir
HBeAg: Hepatitis B e antigen
HBsAg: Hepatitis B surface antigen
HBV: Hepatitis B virus
HCC: Hepatocellular carcinoma
IFN: Interferon
NUC: Nucleos(t)ide analog
OR: Odds ratio
